# Development and validation of a prediction model for refeeding syndrome in ICU patients receiving mechanical ventilation and enteral nutrition support: a single-center retrospective study from China

**DOI:** 10.3389/fmed.2026.1692124

**Published:** 2026-03-05

**Authors:** Nan Feng, Meiying Piao, Mengying Qi, Wenjie Xiao, Wenjuan Wang, Yuju Qin, Haigang Zhang

**Affiliations:** Department of Critical Care Medicine, Shenzhen Nanshan District People's Hospital, Shenzhen, Guangdong, China

**Keywords:** clinical outcomes, ICU, LASSO regression, nomogram, nutritional support, prediction model, refeeding syndrome, risk factors

## Abstract

**Objective:**

To develop and validate a risk prediction model for refeeding syndrome (RFS) in mechanically ventilated patients in the intensive care unit (ICU) receiving initial enteral nutrition therapy.

**Design:**

A retrospective cohort study was conducted at a tertiary hospital in Shenzhen, China.

**Setting:**

This single-center study was conducted in a tertiary hospital in Shenzhen, China.

**Participants:**

Patients who were admitted to the ICU of a tertiary hospital in Shenzhen for the first time and received enteral nutrition support between January 2022 and December 2024 were selected. The cohort was divided into a modeling set (*n* = 664) and a validation set (*n* = 284).

**Methods:**

Factors potentially associated with refeeding syndrome (RFS) were collected, including patients’ clinical indicators and refeeding-related conditions. Patients were divided into RFS and non-RFS groups according to the presence or absence of RFS. Potential variables were screened using the least absolute shrinkage and selection operator (LASSO) regression, followed by multivariate logistic regression analysis; a nomogram model was then constructed and validated.

**Results:**

Among the 664 patients in the modeling cohort, 300 cases (45.18%) developed refeeding syndrome (RFS). Following LASSO regression, multivariate logistic regression analysis was performed, and the results revealed that age ≥ 60 years, Nutritional Risk Screening 2002 (NRS-2002) score ≥ 3 points, Sequential Organ Failure Assessment (SOFA) score ≥ 10 points, Acute Physiology and Chronic Health Evaluation II (APACHE II) score ≥ 20 points, and pre-feeding albumin (ALB) < 30 g/L were identified as independent risk factors for RFS in mechanically ventilated ICU patients receiving enteral nutrition support (*p* < 0.05). Results of receiver operating characteristic (ROC) curve analysis demonstrated that the area under the curve (AUC) for predicting RFS risk in mechanically ventilated ICU patients was 0.859 (95% confidence interval [95% CI]: 0.815–0.903) in the modeling cohort and 0.832 (95% CI: 0.802–0.862) in the validation cohort. Calibration curve analysis showed that the predicted curves of both the modeling and validation cohorts were in good agreement with the ideal curve.

**Conclusion:**

The prediction model demonstrates good discrimination and calibration, enabling intuitive and convenient identification of ICU patients receiving enteral nutrition who are at high risk of refeeding syndrome, thereby providing a reference for early screening and intervention.

## Highlights

- This study successfully developed and validated a predictive model for refeeding syndrome (RFS) risk in ICU patients after their first feeding.
- The model is clinically valuable, aiding early RFS risk identification and enabling personalized nutrition support plans to reduce RFS incidence and improve patient outcomes.
- To some extent, the convenience sampling used in this study weakened the results.
- Though incorporating established prognostic factors for refeeding syndrome (RFS) may have led to other risk factors being missed.

## Introduction

Refeeding syndrome (RFS) is a metabolic disorder that may occur after restarting nutritional support in malnourished or chronically starved patients, typically within 72 h of feeding initiation. It is characterized by electrolyte imbalances, such as hypophosphatemia, hypokalemia, and hypomagnesemia, as well as clinical manifestations, including arrhythmias, respiratory failure, and altered consciousness (1–3). ICU patients, due to critical illness, poor nutritional status, and high metabolic demands, are at a heightened risk for RFS. The incidence of RFS ranges from 17.3 to 59.4%, with a 6-month mortality rate of up to 33.9% (4). The core pathophysiological mechanism is the rapid shift in metabolic state from catabolism to anabolism triggered by an abrupt surge in enteral nutrition-induced insulin; chronic malnutrition downregulates insulin secretion and increases insulin resistance, while the reintroduction of carbohydrates stimulates the pancreas to abruptly release insulin (5). This process leads to the intracellular redistribution of electrolytes, predominantly hypophosphatemia, hypokalemia, and hypomagnesemia. Phosphorus is driven into cells to participate in adenosine triphosphate (ATP) synthesis, potassium is involved in glycogen synthesis, and magnesium acts as a cofactor for enzymatic reactions. Hypophosphatemia, the hallmark manifestation of RFS, can cause impaired cellular energy metabolism, respiratory muscle weakness, arrhythmias, and neurological abnormalities, all of which are severe complications in ICU patients with impaired cardiopulmonary function. Concomitant fluid retention and sodium overload further exacerbate organ dysfunction, which is particularly prominent in patients with underlying cardiac or renal diseases (3, 4). RFS not only exacerbates metabolic disturbances but may also lead to multi-organ dysfunction, prolonged hospitalization, and increased mortality risk (6, 7).

Currently, there is no global consensus regarding the definition of RFS. In 2020, the American Society for Parenteral and Enteral Nutrition (ASPEN) defined RFS as the development of hypophosphatemia, hypokalemia, hypomagnesemia (one or more), or manifestations of thiamine deficiency shortly after initiating caloric intake in malnourished patients (within hours to days) (8). The nonspecific clinical features of RFS, which overlap with those of multi-organ dysfunction syndrome, are often masked by critical illness (9–11). Additionally, insufficient awareness and preventive strategies among healthcare providers have hindered the comprehensive assessment of its risk factors, underscoring the urgent need for rapid and accessible early warning tools. Therefore, the development and validation of a risk prediction model for RFS in ICU patients are critical for providing evidence-based guidance for early prevention.

## Methods

### Design

This single-center retrospective cohort study was conducted at a tertiary hospital in Shenzhen, China. This study followed the Strengthening the Reporting of Observational Studies in Epidemiology statement for reporting observational studies in epidemiology.

### Setting

This study was conducted in a tertiary care hospital, and the departments involved in the work included the emergency ward and intensive care unit.

This work was supported by a grant from the Shenzhen Nanshan District Health Bureau (Project Number NS2024074). Patients who were admitted to the ICU and received nutritional support between January 2022 and December 2024 were selected using convenience sampling.

### Study participants

A total of 948 ICU patients were recruited from a tertiary hospital in Shenzhen. Of these, 664 patients (70%) admitted between January 2022 and December 2023 were allocated to the modeling set, and 284 patients (30%) admitted between January 2024 and December 2024 comprised the validation set. Inclusion criteria: ① Age ≥ 18 years, ② ICU length of stay ≥ 48 h, ③ Received exclusive enteral nutrition for > 72 h with available blood phosphorus results both at admission and within 72 ± 12 h of initiating enteral nutrition. Exclusion criteria: ① Age > 85 years or < 18 years, ② Pre-existing severe electrolyte disturbances before ICU admission, diseases affecting electrolyte metabolism, such as chronic kidney disease or liver cirrhosis, ③ Diagnosis of malignant tumors or receiving radiotherapy/chemotherapy, ④ Pregnancy or lactation, ⑤ incomplete data, ⑥ Recent parathyroidectomy, renal replacement therapy, use of phosphate-binding agents, or undergoing therapeutic hypothermia, ⑦ Patients with a history of anorexia nervosa, a medication history, and/or excessive alcohol consumption ⑧ received bariatric/metabolic surgery. This study was approved by the Scientific Research Ethics Committee of Shenzhen Nanshan People’s Hospital (Approval No.: Nanshan Hospital Research Ethics [2025] 0317032).

### Enteral nutritional support protocol

Recommended nutritional support strategy: Phased initiation of enteral nutrition with low-calorie supply (10–20 kcal/kg/day) with a gradual escalation of energy delivery to the preset target requirements over 3–7 days, based on the patient’s enteral nutrition tolerance and clinical status.

### Sample size calculation

According to the calculation formula for estimating population rates through sampling surveys, n = Z^2^_*α*/2_ P(1–P)/*δ*^2^, assuming an error allowance of δ = 0.05 and an RFS incidence rate of approximately 17.3–59.4% (mean, 38.35%), α = 0.05, Z_0.05/2_ = 1.96, and *p* = 0.384, the calculated sample size was *n* = 363. Considering the exclusion criteria, a dropout rate of 10% was set, and the required sample size was determined to be ≥400.

### Research instruments

#### General information questionnaire

Collected demographic data (age, sex, weight), laboratory results (serum phosphorus, potassium, magnesium, albumin, prealbumin), nutritional support protocol (initiation time, route, dosage), length of hospital/ICU stay, duration of mechanical ventilation, and 28-day mortality.

#### Nutritional risk screening scale (NRS 2002)

This scale comprises three domains: age score: 1 point for age >70 years, 0 points for ≤70 years. Nutritional status score: ranges from 0 to 3 points. Disease severity score: Ranges from 0 to 3 points. The total scores range from 0 to 7, with a score ≥3 indicating malnutrition risk. The scale demonstrates good reliability (Cronbach’s *α* = 0.88) and content validity of 0.79 (12). In this study, this scale was assessed by clinical dietitians from the Department of Clinical Nutrition.

#### Acute Physiology and Chronic Health Evaluation (APACHE II)

This scale includes three parts: acute physiology, age, and chronic health scores, with a total score of 71 points. A higher score indicates a more critical condition (13). It is reliable in most ICU settings, with a sensitivity and specificity of 85.3 and 79%, respectively (14). In this study, this scale was assessed by physicians.

#### Sequential Organ Failure Assessment (SOFA) scale

This scale assesses the degree of organ dysfunction or failure and predicts the prognosis of critically ill patients. It covers six aspects: respiration, cardiovascular, liver, kidney, nervous system, and coagulation, and has a total score of 0–48. A higher score indicates a more severe condition (15). The sensitivity and specificity for predicting ICU mortality are 73.6 and 70.4%, respectively (16). In this study, this scale was assessed by physicians.

### Diagnostic criteria

Referring to domestic scholars’ views and the 2020 ASPEN consensus on RFS definition and diagnosis (8), in this study, RFS is defined as the occurrence within 3 days of starting nutritional support of serum phosphorus <0.85 mmol/L with a ≥ 30% decrease from baseline or an absolute reduction ≥0.16 mmol/L; concurrent electrolyte abnormalities: hypokalemia (serum potassium <3.50 mmol/L) and hypomagnesemia (serum magnesium <0.75 mmol/L); and clinical manifestations: presence of at least one multisystem symptom or sign, including tachycardia, respiratory failure, arrhythmia, nausea/vomiting, edema, delirium, or coma.

### Data collection

Two nursing graduate students, trained by ICU physicians and certified, were proficient in RFS and nutrition assessment. During data collection, they strictly selected participants based on the inclusion/exclusion criteria, gathering clinical data via electronic and paper records, and excluding ineligible cases. All data were double-checked and entered by two people.

### Statistical analysis

Data analysis was performed using SPSS 27.0 and RStudio software. Measurement data are expressed as mean ± standard deviation (x ± s). For normally distributed measurement data, comparisons between groups were conducted using an independent-sample *t*-test. Count data are presented as cases (percentages), and intergroup comparisons were performed using the chi-square test or Fisher’s exact test. Comparisons of ordinal data or non-normally distributed measurement data were performed using the rank-sum test. LASSO regression was applied to screen for important predictive factors, and multivariate Logistic regression analysis was performed to identify the independent influencing factors for refeeding syndrome (RFS) development in mechanically ventilated ICU patients receiving enteral nutrition support. A nomogram model for predicting RFS risk was constructed using RStudio software. Internal validation of the nomogram model was conducted via 1,000 repeated resamplings with the bootstrap method, while external validation was performed using data from the external validation set. The ROC curve was used to evaluate the predictive value of the nomogram model for RFS development in mechanically ventilated ICU patients receiving enteral nutrition support in both the modeling and external validation sets. The Hosmer–Lemeshow goodness-of-fit test and calibration curve analysis were adopted to assess the goodness of fit of the nomogram model in the modeling and external validation sets. A two-sided *p*-value < 0.05 was considered statistically significant.

### Patient and public involvement

Patients and/or the public were not involved in the design, conduct, reporting, or dissemination of this study.

## Results

### Comparison of general data

**Figure 1:**
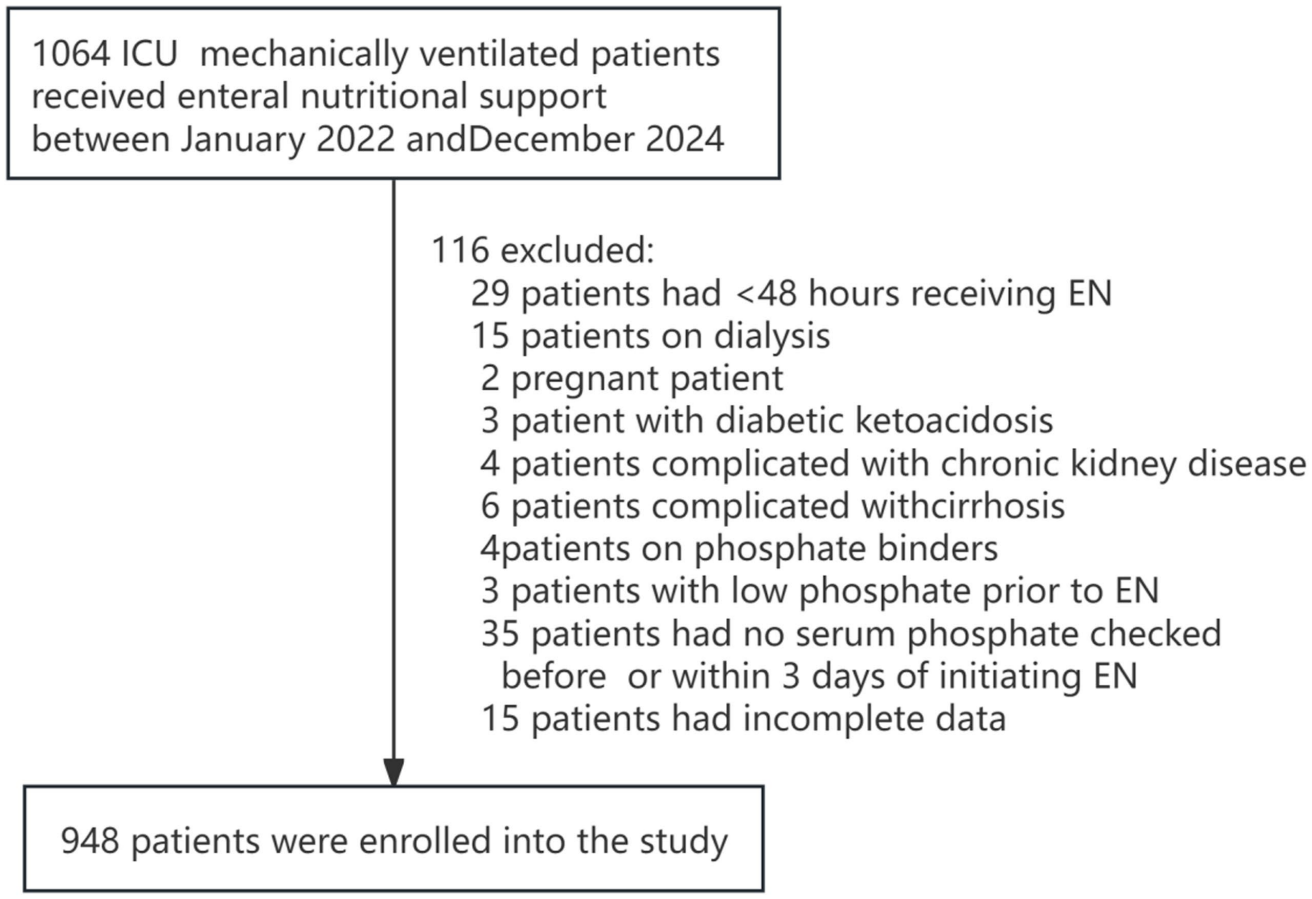
Flowchart of patient enrollment. EN, Enteral nutritional.

A total of 1,064 ICU patients received enteral nutrition support therapy, with 948 patients ultimately included in this study (Figure 1). The cohort comprised 535 males and 413 females, with a mean age of 67.81 ± 19.42 years. Refeeding syndrome (RFS) was identified in 421 patients (44.40%), including 300 cases (45.18%) in the modeling set and 121 cases (42.61%) in the validation set, with no statistically significant difference in detection rates between the two groups (χ^2^ = 0.53, *p* = 0.96).

In the modeling set, the RFS group exhibited significantly longer total hospitalization duration, intensive care unit (ICU) stay, and mechanical ventilation time than the non-RFS group (*p* < 0.05). Additionally, the RFS group showed significantly higher NRS2002 nutritional risk screening and APACHE II scores than the non-RFS group (*p* < 0.05) (Table 1).

**Table 1:** Demographic characteristics and prognostic outcomes of patients.

Variable		non-RFS group (*n* = 364)	RFS group (*n* = 300)	t/χ2 test	*p* value
Age (years, mean ± SD)		59.32 ± 19.15	78.12 ± 14.18	−14.535	<0.001*
Gender [*n* (%)]	Male	203 (55.77%)	177 (59%)	0.701	0.402
	Female	161 (44.23%)	123 (41%)		
Death within 28 days [*n* (%)]	No	345 (94.78%)	215 (71.67%)	66.512	<0.001*
	Yes	19 (5.22%)	85 (28.33%)		
BMI (kg/m^2,^ mean ± SD)		22.58 ± 4.05	20.46 ± 3.53	7.209	<0.001*
Pre-feeding magnesium (mmol/L, mean ± SD)		0.82 ± 0.18	0.85 ± 0.15	−1.865	0.063
Pre-feeding potassium (mmol/L, mean ± SD)		3.93 ± 0.55	3.99 ± 0.45	−1.470	0.142
Pre-feeding phosphorus (mmol/L, mean ± SD)		1.02 ± 0.29	1.05 ± 0.38	−1.331	0.184
Phosphorus within 24 h post-feeding (mmol/L, mean ± SD)		0.92 ± 0.24	0.83 ± 0.30	4.172	<0.001*
Phosphorus within 48 h post-feeding (mmol/L, mean ± SD)		0.93 ± 0.26	0.85 ± 0.26	3.994	<0.001*
Phosphorus within 72 h post-feeding (mmol/L, mean ± SD)		0.96 ± 0.23	0.86 ± 0.22	5.709	<0.001*
APACHEII Score (mean ± SD)		18.87 ± 8.00	27.51 ± 8.53	−13.456	<0.001*
NRS2002 Score (mean ± SD)		3.19 ± 1.86	4.91 ± 1.53	−13.085	<0.001*
Mechanical ventilation time (days, mean ± SD)		3.23 ± 5.25	12.36 ± 40.78	−4.237	<0.001*
ICU Stay (days, mean ± SD)		8.84 ± 7.30	12.72 ± 29.25	−2.242	0.026*
Total hospitalization duration (days, mean ± SD)		10.78 ± 14.59	16.55 ± 45.95	−2.090	0.037*

Numbers marked with * are significant with a *p*-value <0.05.

Among the 664 patients in the modeling cohort, 300 (45.18%) developed RFS. Age ≥60 (*χ*^2^ = 134.168, *p* < 0.001), NRS 2002 score ≥3 (*χ*^2^ = 68.368, *p* < 0.001), APACHE II score ≥20 (*χ*^2^ = 114.909, *p* < 0.001), SOFA score ≥10 (*χ*^2^ = 28.900, *p* < 0.001), BMI ≤ 18.5 kg/m^2^ (*χ*^2^ = 7.991, *p* < 0.005), pre-feeding albumin <30 g/L (*χ*^2^ = 45.476, *p* < 0.001), pneumonia (*χ*^2^ = 31.195, *p* < 0.001), COPD comorbidity (*χ*^2^ = 5.546, *p* = 0.019), pre-feeding transfusion (*χ*^2^ = 9.182, *p* = 0.002), pre-feeding hemoglobin (*χ*^2^ = 5.269, *p* < 0.001), and starting feeding within 48 h of ICU admission (*χ*^2^ = 4.606, *p* = 0.032) were significantly associated with RFS (*p* < 0.05) (Table 2).

**Table 2:** Comparison of factors between the RFS and non-RFS groups.

Variable		RFS group (*n* = 300)	Non-RFS group (*n* = 364)	t/χ2 test	*p* value
Age [*n* (%)]	<60	28 (9.33)	188 (51.65)	134.168	<0.001*
	≥60	272 (90.67)	176 (48.35)		
NRS2002 Score [*n* (%)]	<3	20 (6.67)	120 (32.97)	68.368	<0.001*
	≥3	280 (93.33)	244 (67.03)		
APACHEIIScore [*n* (%)]	<20	52 (17.22)	212 (58.24)	114.909	<0.001*
	≥20	248 (82.67)	152 (41.76)		
Pre-feeding ALB [*n* (%)]	<30	172 (57.33)	296 (81.32)	45.476	<0.001*
	≥30	128 (42.67)	68 (18.68)		
Tracheal intubation [*n* (%)]	No	136 (45.33)	188 (51.64)	2.625	0.105
	Yes	164 (54.67)	176 (48.35)		
SOFA Score [*n* (%)]	<10	180 (60.00)	288 (79.12)	28.900	<0.001*
	≥10	120 (40.00)	76 (20.88)		
BMI [*n* (%)]	<18.5	80 (26.67)	64 (17.58)	7.991	0.005*
	≥18.5	220 (73.33)	300 (82.42)		
Severe pneumonia [*n* (%)]	No	116 (38.67)	220 (60.44)	31.190	<0.001*
	Yes	184 (61.33)	144 (39.56)		
MODS [*n* (%)]	No	280 (93.33)	324 (89.01)	3.738	0.053
	Yes	20 (6.67)	40 (10.99)		
Renal failure [*n* (%)]	No	200 (66.67)	256 (70.33)	1.026	0.311
	Yes	100 (33.33)	108 (29.67)		
Diabetes [*n* (%)]	No	252 (84.00)	324 (89.01)	3.592	0.058
	Yes	48 (16.00)	40 (10.99)		
Pre-feeding diuretic use [*n* (%)]	No	164 (54.67)	208 (57.14)	0.409	0.522
	Yes	136 (45.33)	156 (42.86)		
Pre-feeding insulin use [*n* (%)]	No	184 (61.33)	232 (63.74)	0.406	0.524
	Yes	116 (38.67)	132 (36.26)		
Pre-feeding transfusion [*n* (%)]	No	188 (62.67)	268 (73.63)	9.182	0.002*
	Yes	112 (37.33)	96 (26.37)		
Feeding started within 48 h of ICU admission [*n* (%)]	No	224 (74.67)	244 (67.03)	4.606	0.032*
	Yes	76 (25.33)	120 (32.97)		
Enteral nutrition route [*n* (%)]	Gastric tube	264 (88.00)	320 (87.91)	0.001	0.972
	Nasointestinal tube	36 (12.00)	44 (12.09)		
COPD [*n* (%)]	No	248 (82.67)	324 (89.01)	5.546	0.019*
	Yes	52 (17.33)	40 (10.99)		
Gastrointestinal decompression before feeding [*n* (%)]	No	204 (68.00)	256 (70.33)	0.419	0.517
	Yes	96 (32.00)	108 (29.67)		
Fasting time		56.25 ± 53.01	56.23 ± 64.64	−0.005	0.996
Daily intake (ml, mean± SD)		794.96 ± 78.58	850.10 ± 79.76	1.865	0.063
Feeding rate (ml/h, mean± SD)		39.52 ± 17.37	49.11 ± 61.57	2.838	0.005*
Pre-feeding Hb (g/L, mean± SD)		96.85 ± 19.44	105.63 ± 23.47	5.269	<0.001*

Numbers marked with * are significant with a *p*-value <0.05.

BMI, body mass index; ALB, albumin; Hb, hemoglobin; COPD, chronic obstructive pulmonary disease.

### Screening of potential factors for RFS

**Figure 2:**
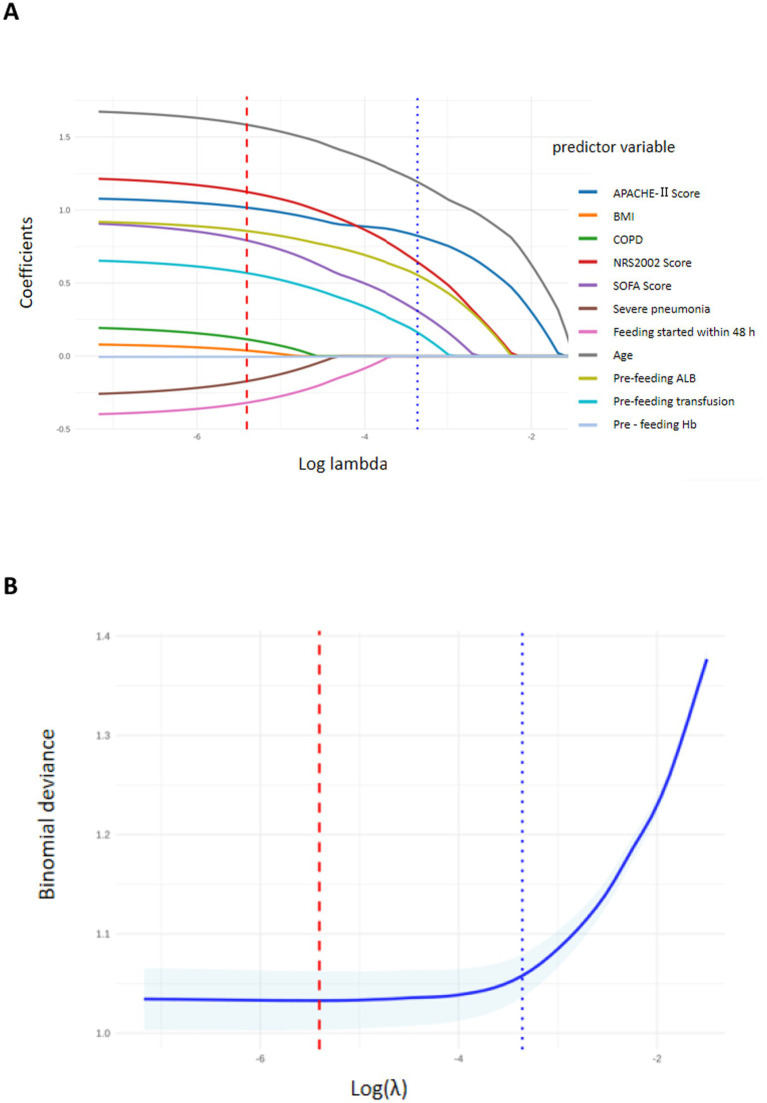
Clinical feature selection using LASSO regression. **(A)** LASSO coefficients for 11 clinical features. **(B)** Ten-fold cross-validation of the LASSO regression model.

With the occurrence of RFS as the dependent variable, LASSO regression analysis was performed to screen 11 potential influencing factors, and ten-fold cross-validation was used to determine the optimal lambda (*λ*) value. As the penalty coefficient λ increased, the coefficients of the independent variables were gradually decreased (Figure 2A). Ultimately, the λ value with the minimum error in the ten-fold cross-validation (λ = 0.004) was selected as the optimal value (Figure 2B). Six important predictive factors were identified, including age ≥ 60 years, Nutritional Risk Screening 2002 (NRS-2002) score ≥ 3 points, Sequential Organ Failure Assessment (SOFA) score ≥ 10 points, Acute Physiology and Chronic Health Evaluation II (APACHE II) score ≥ 20 points, pre-feeding albumin (ALB) < 30 g/L, and pre-feeding blood transfusion.

### Multivariate analysis of RFS

The potential variables screened by LASSO regression were included as independent variables for multivariate logistic regression analysis, and the results showed that age ≥ 60 years, Nutritional Risk Screening 2002 (NRS-2002) score ≥ 3 points, Sequential Organ Failure Assessment (SOFA) score ≥ 10 points, Acute Physiology and Chronic Health Evaluation II (APACHE II) score ≥ 20 points, pre-feeding albumin (ALB) < 30 g/L, and blood transfusion were independent influencing factors for the development of RFS in mechanically ventilated ICU patients receiving enteral nutrition support (*p* < 0.05), as shown in Table 3.

**Table 3:** Multivariate analysis of RFS.

Variable	B	SE	Wald	*p* value	OR (95%CI)
Age	1.630	0.266	6.14	<0.001	5.103 (3.033–8.587)
APACHE-II Score	1.059	0.216	4.91	<0.001	2.883 (1.889–4.340)
NRS2002 Score	1.182	0.302	3.91	<0.001	3.262 (1.805–5.900)
SOFA Score	0.745	0.210	3.55	<0.001	2.107 (1.396–3.181)
Pre-feeding transfusion	0.607	0.210	2.89	0.004	1.835 (1.216–2.768)
Pre-feeding ALB	0.916	0.210	4.37	<0.001	2.498 (1.656–3.768)
Constant	−3.777	0.3676	−10.27	<0.001	–

### Construction of a nomogram model for predicting RFS risk

**Figure 3:**
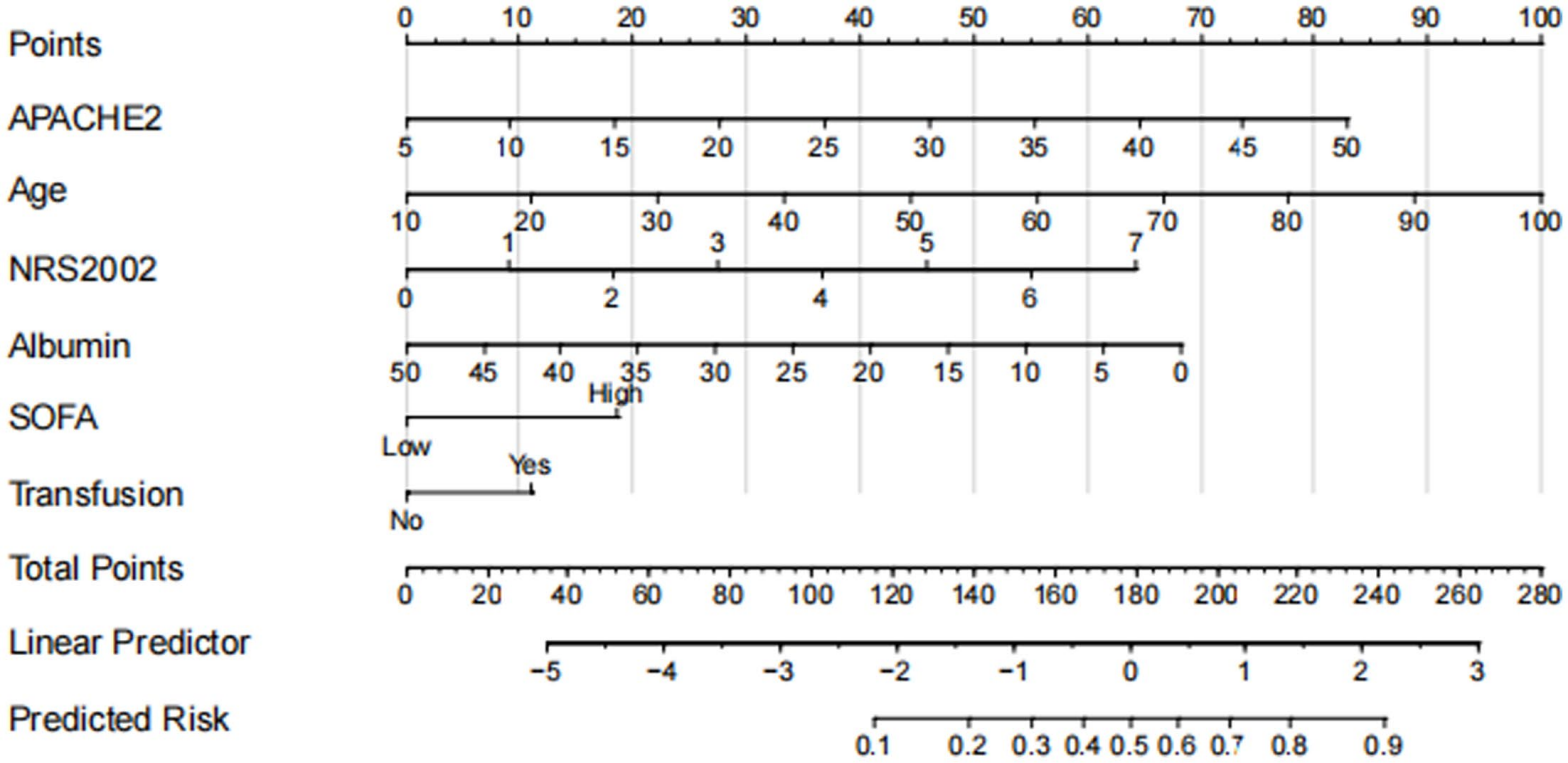
Nomogram for predicting the risk of refeeding syndrome (RFS) in ICU patients.

Based on the results of the multivariate analysis, a nomogram model for predicting the risk of RFS in mechanically ventilated ICU patients receiving enteral nutrition support was constructed (Figure 3).

### Internal validation

**Figure 4:**
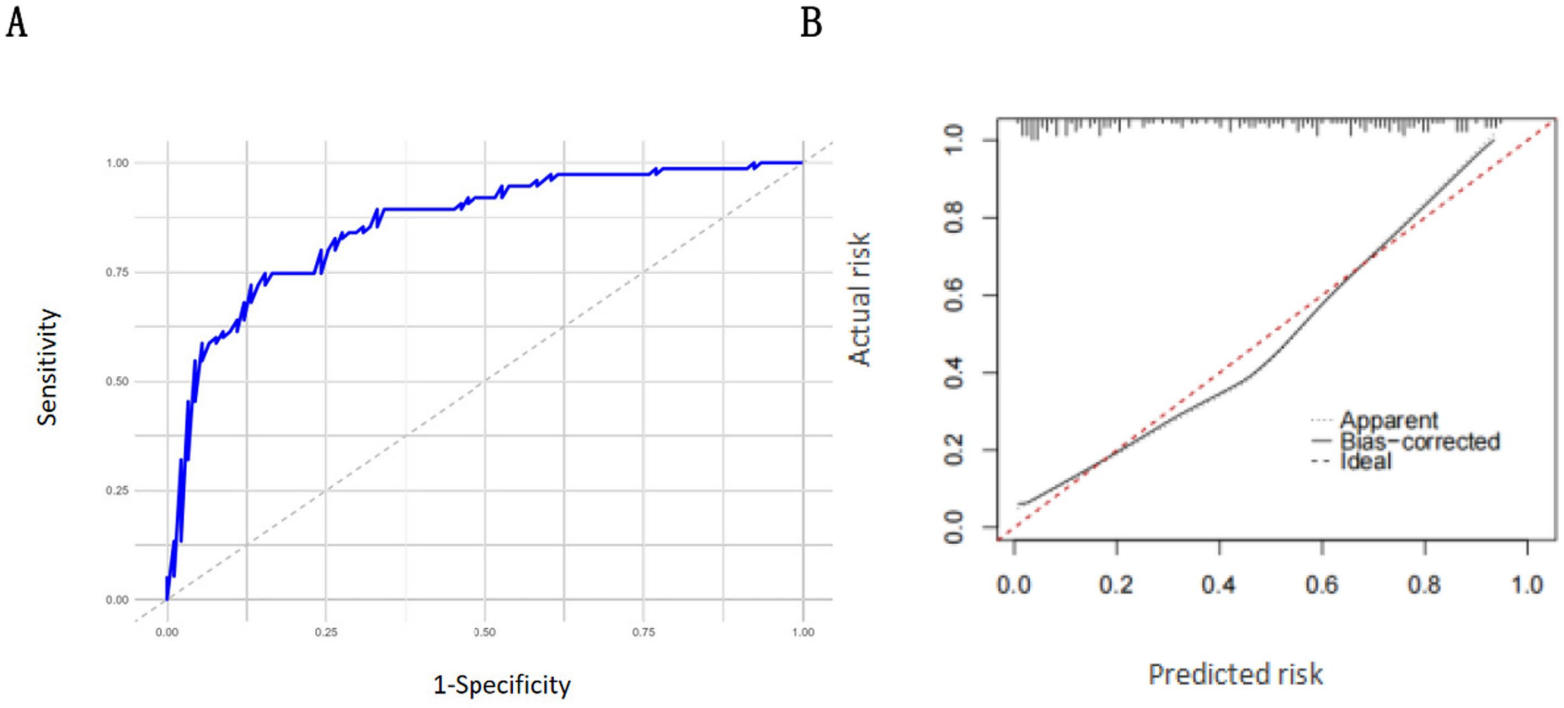
Internal verification of the nomogram model: **(A)** ROC curve of the modeling set; **(B)** calibration curve of the modeling set.

The results of the receiver operating characteristic (ROC) curve analysis showed that the area under the curve (AUC) of the nomogram model for predicting RFS development in mechanically ventilated ICU patients receiving enteral nutrition support was 0.859 (95% CI: 0.815–0.903) (Figure 4A). The results of the Hosmer–Lemeshow goodness-of-fit test and calibration curve analysis demonstrated that the nomogram model had a good fit in the modeling cohort (Figure 4B).

### External validation

**Figure 5:**
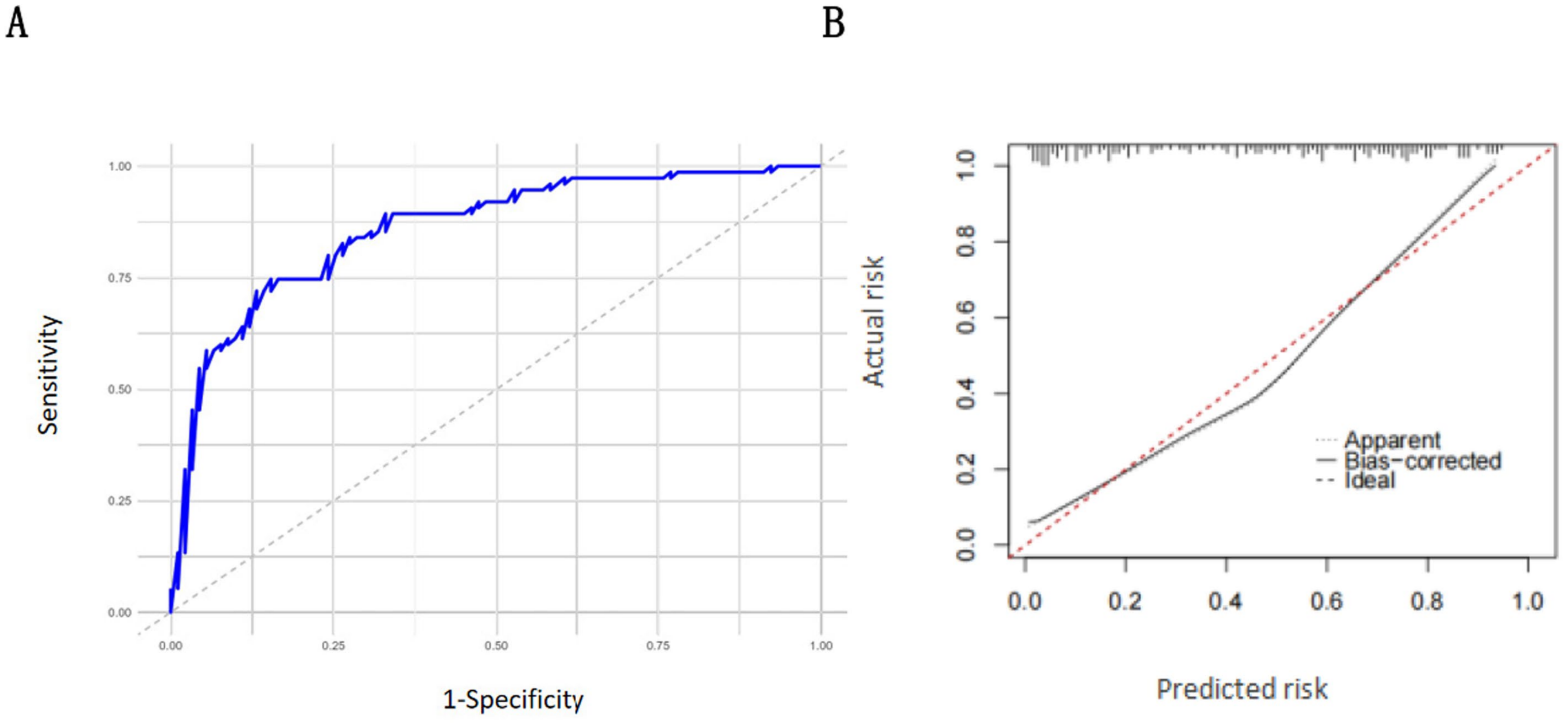
External verification of the nomogram model: **(A)** ROC curve of the validation set; **(B)** calibration curve of the validation set.

The results of receiver operating characteristic (ROC) curve analysis showed that the area under the curve (AUC) of the nomogram model for predicting RFS development in mechanically ventilated ICU patients receiving enteral nutrition support was 0.832 (95% CI: 0.802–0.862) in the validation cohort (Figure 5A). The results of the Hosmer–Lemeshow goodness-of-fit test and calibration curve analysis demonstrated that the nomogram model had a good fit in the validation cohort (Figure 5B).

**Figure 6:**
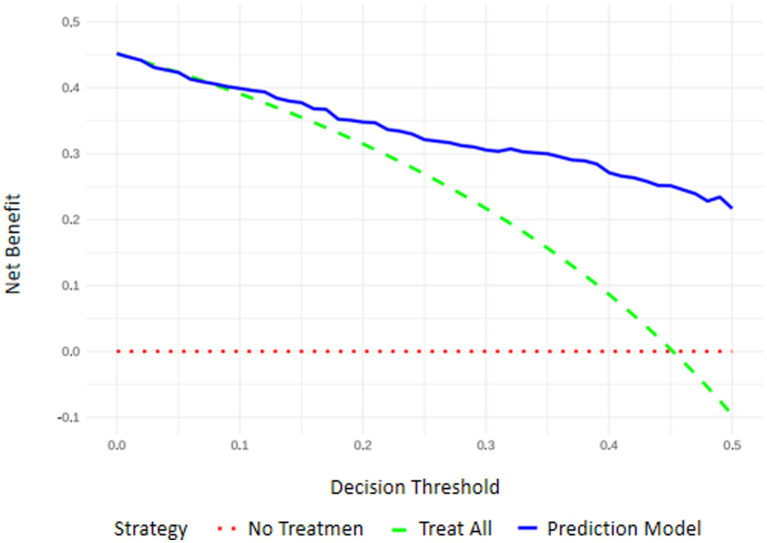
Schematic diagram of decision curve analysis.

Results of decision curve analysis (DCA) showed that the net benefit rate of patients was > 0 when the threshold probability of the nomogram for predicting RFS risk in mechanically ventilated ICU patients ranged from 0.10 to 0.90 (Figure 6).

## Discussion

### Risk prediction model

The results of this study indicate that the detection rate of RFS among ICU patients receiving enteral nutrition therapy was 44.4%. RFS can lead to prolonged mechanical ventilation, longer hospital stays, and even death, which is consistent with previous findings (17–19). Thus, building a risk prediction model for early RFS identification and timely intervention is crucial. RFS lacks specific clinical symptoms. Some studies suggest that advanced age, high SOFA scores, fasting over two days, craniocerebral surgery, starting nutrition within 48 h of ICU admission, short continuous infusion time, excessive protein intake, pre-feeding serum albumin levels, chemotherapy, and the use of acid suppressants, diuretics, and insulin are associated with RFS risk (7, 20, 21). The National Institute for Health and Care Excellence (NICE) guidelines identify RFS risk factors such as BMI, involuntary weight loss, starvation, alcohol abuse history, and pre-feeding serum phosphorus, potassium, and magnesium levels. However, Wong et al. found that the NICE guidelines have poor predictive ability (22), likely due to the lack of unified RFS diagnostic criteria and numerous influencing factors (2). In this study, based on the ESPEN guidelines, relevant research, ASPEN consensus, and current patient conditions, 22 factors potentially related to RFS in ICU patients were chosen. Through univariate and binary logistic regression analyses, six indicators were selected: age, NRS-2002 score, SOFA score, Acute Physiology and APACHE II score, pre-feeding albumin (ALB) level, and whether pre-feeding transfusion occurred. A visual nomogram was developed for RFS risk prediction. The model showed good predictive ability in both the modeling (C-statistic: 0.854) and validation sets (C-statistic: 0.859). The calibration curves indicated no significant differences between the predicted and observed values. This suggests that the model has sufficient discriminatory power and clinical effectiveness for predicting RFS risk. The six predictors in the model are common in ICU settings and primarily involve assessments with easily obtainable data and no additional costs. By referring to the nomogram, ICU healthcare providers can calculate the total score of the six predictive factors to obtain the predicted risk value of RFS in patients receiving enteral nutrition therapy. Using the optimal cutoff of 0.599, high- and low-risk groups can be distinguished, enabling early RFS screening and avoiding unnecessary resource wastage. For example, for an 80-year-old ICU patient with an NRS-2002 score of 6, SOFA score over 10, APACHE II score of 25, pre-feeding ALB of 30 g/L, and no pre-feeding transfusion, the nomogram total score was 216, indicating an RFS risk of 0.78. This patient would be considered high-risk and should receive early intervention.

In a study by Wong et al. (22), the incidence of refeeding hypophosphatemia (RH) ranged from 23 to 48% depending on the diagnostic criteria applied. The guidelines developed by the National Institute for Health and Clinical Excellence (NICE) exhibited poor discriminative ability across all diagnostic criteria, with an area under the receiver operating characteristic (ROC) curve of only 0.43–0.53. The study confirmed that critical illness, history of diuretic use, and hypomagnesemia prior to parenteral nutrition (PN) administration were independent risk factors for RH, which is consistent with the variable selection strategy adopted in our study. However, most of these models are limited by small sample sizes and a lack of external validation.

### Application of assessment tools in early screening

This study demonstrated that patients with an NRS-2002 score ≥3 had a significantly increased risk of RFS (odds ratio [OR] = 3.548, *p* < 0.001). The NRS-2002 is a validated tool for assessing nutritional risk, and its score directly reflects the patient’s nutritional status. Higher NRS-2002 scores indicate a more severe malnutrition risk. Such patients often struggle to adapt to abrupt increases in nutrient intake during nutritional support, thereby predisposing them to RFS. The NRS-2002 comprehensively evaluates nutritional risk by incorporating multiple dimensions, including weight change, dietary intake, and disease severity (23). In ICU patients, who frequently experience complex critical illnesses and high rates of malnutrition, NRS-2002 is particularly valuable for identifying high-risk individuals for RFS, enabling the early implementation of targeted preventive measures (24). These findings align with the emphasis on “early nutritional risk assessment” in international guidelines (ESPEN, 2023) (25).

The results further identified SOFA score ≥10 and APACHE II score ≥20 as significant predictors of RFS (OR = 2.519 and 2.824, respectively), consistent with the findings of Wei Zhang et al. (26) in neurocritical care patients. The SOFA score reflects organ dysfunction severity, guiding clinicians in determining tolerance to nutritional support and selecting appropriate modalities. The APACHE II score evaluates global nutritional risk and metabolic status, aiding in optimizing the timing, dosage, and composition of nutritional interventions (27). Integrating the SOFA and APACHE II scores allows precision-guided clinical decision-making. By synthesizing organ function, metabolic demands, and nutritional risks, clinicians can develop individualized nutritional support strategies to mitigate complications, such as RFS, and enhance patient recovery.

### Risk factors for RFS

Patients with a history of blood transfusion exhibited a 1.994-fold increased risk of refeeding syndrome (RFS) (OR = 1.994, *p* < 0.05). Transfusion may trigger immune and inflammatory responses, including the release of inflammatory mediators from stored red blood cells (e.g., TNF-*α* ↑200 ng/mL, and IL-1β ↑150 pg./mL) and immunosuppression (e.g., CD4+/CD8 + ratio ↓1.8-fold), which can promote intestinal pathogen overgrowth (28). Additionally, massive transfusion may disrupt electrolyte homeostasis (e.g., potassium and phosphorus), aligning with the pathophysiological mechanisms of RFS (29). Moreover, transfusion recipients often present with critical illness, malnutrition, or multi-organ dysfunction, collectively exacerbating RFS risk. Incorporating transfusion history into risk prediction models enhances comprehensive risk stratification, particularly for patients requiring large-volume transfusions due to acute blood loss, who warrant heightened vigilance for RFS.

Hypoalbuminemia emerged as a significant predictor of RFS (OR = 2.526). Lower serum albumin (ALB) levels typically reflect malnutrition or impaired hepatic synthetic function (30). Reduced ALB compromises nutritional reserve capacity and diminishes tolerance to nutritional support (31). During refeeding, insufficient protein reserves may precipitate metabolic disturbances and electrolyte imbalances characteristic of RFS (32). Therefore, pre-feeding ALB monitoring is critical for assessing RFS risk and guiding clinical interventions.

### Management of RFS in critically ill patients

Management of RFS emphasizes proactive risk stratification, early intervention, and individualized nutritional titration, aligned with the latest guidelines of the European Society for Clinical Nutrition and Metabolism (ESPEN) (25). Key strategies include: (1) pre-refeeding assessment of malnutrition severity (using tools such as the GLIM criteria) and baseline electrolyte monitoring (phosphate, potassium, magnesium, calcium) (33); (2) phased nutritional initiation with low-calorie EN (10–20 kcal/kg/day) in high-risk patients, gradually increasing to target energy requirements over 3–7 days (34); (3) prophylactic and corrective electrolyte supplementation (oral or intravenous) guided by serial monitoring (at least daily for the first 5 days, or more frequently in severe cases) (11); (4) prioritization of protein intake (1.2–2.0 g/kg/day) to mitigate muscle catabolism while limiting carbohydrate load (8); (5) close monitoring of fluid balance and hemodynamic status to prevent volume overload. For severe RFS complicated by arrhythmias or respiratory failure (25), intensive care support (e.g., continuous cardiac monitoring, mechanical ventilation) and targeted electrolyte replacement (e.g., IV phosphate for serum phosphate <0.32 mmol/L) are imperative; and (6) Multidisciplinary Team Approach (35): RFS requires a multidisciplinary approach for identification and management. Nursing staff, dietitians, physicians, and pharmacists should collectively participate in screening, monitoring, and managing both the risk and actual RFS (36). Their core role is to ensure that patients receive nutritional support with regular monitoring of serum electrolyte levels and clinical parameters.

### Limitations

While the sample size in this study met the minimum requirements for statistical power, the exclusive recruitment of participants from a single tertiary hospital in Southern China may introduce geographic and healthcare-level biases. These limitations could constrain the model’s external validity, necessitating further multicenter validation across diverse healthcare settings.

Furthermore, our variable selection process, although incorporating established prognostic factors for RFS, may have omitted biologically plausible predictors. Key pathophysiological determinants, such as genetic predisposition, gut microbiota composition, and inflammatory biomarkers (e.g., IL-6, CRP trajectories), have not been systematically evaluated. Emerging evidence suggests that these factors may modulate mitochondrial recovery during refeeding. Future iterations of the model should prioritize integrating multi-omics data to address this mechanistic gap, particularly through collaboration with genomic databases and longitudinal microbiota monitoring cohorts.

## Conclusion

This study successfully developed and validated a risk prediction model for RFS in ICU patients undergoing initial enteral nutrition therapy. The model integrates multiple key factors: age, NRS-2002 scores, SOFA scores, APACHE II scores, pre-feeding ALB levels, and transfusion status. A nomogram model was established using logistic regression and R software. With an AUC of 0.860 (95%CI: 0.832–0.888), the model had a sensitivity of 72.0% and a specificity of 87.9% at the optimal cutoff of 0.599, showing good discrimination and calibration. The C-statistics were 0.854 (95%CI: 0.135–0.169) for internal validation and 0.859 (95%CI: 0.815–0.903) for external validation, with calibration curves and Brier scores confirming a good fit.

The model is clinically valuable, aiding early RFS risk identification and enabling personalized nutrition support plans to reduce RFS incidence and improve patient outcomes. For high-risk patients, cautious strategies, such as gradual nutrient increase and electrolyte monitoring, can prevent or alleviate RFS. Future plans include refining the model and conducting multicenter trials to confirm its effectiveness and feasibility in real-world settings, thereby promoting its widespread clinical use.

## Data Availability

The datasets presented in this study can be found in online repositories. The names of the repository/repositories and accession number(s) can be found in the article/supplementary material.
